# Telomere Length: A Potential Biomarker for the Risk and Prognosis of Stroke

**DOI:** 10.3389/fneur.2019.00624

**Published:** 2019-06-13

**Authors:** Yanjun Tian, Shuai Wang, Fengjuan Jiao, Qingsheng Kong, Chuanxin Liu, Yili Wu

**Affiliations:** ^1^Collaborative Innovation Center for Birth Defect Research and Transformation of Shandong Province, Jining Medical University, Jining, China; ^2^Shandong Collaborative Innovation Center for Diagnosis, Treatment and Behavioral Interventions of Mental Disorders, Institute of Mental Health, Jining Medical University, Jining, China; ^3^Shandong Key Laboratory of Behavioral Medicine, School of Mental Health, Jining Medical University, Jining, China

**Keywords:** telomere, telomerase, telomere length, risk of stroke, prognosis of stroke

## Abstract

Stroke is one of the leading causes of death and disability worldwide. Age is associated with increased risk of stroke, while telomere length shortening plays a pivotal role in the process of aging. Moreover, telomere length shortening is associated with many risk factors of stroke in addition to age. Accumulated evidence shows that short leukocyte telomere length is not only associated with stroke occurrence but also associated with post-stroke recovery in the elderly population. In this review, we aimed to summarize the association between leukocyte telomere length and stroke, and discuss that telomere length might serve as a potential biomarker to predict the risk and prognosis of stroke.

## Introduction

Stroke is an acute cerebrovascular disease caused by the disruption of blood supply. As the second leading cause of death and the third leading cause of disability worldwide (1), stroke brings heavy burden to both family and health care system. The incidence of stroke is increased with age and ~65% of stroke cases occur in individuals older than 65 years. In addition to age *per se*, various risk factors of stroke are age-related, including hypertension, atherosclerosis, atrial fibrillation, high cholesterol, and diabetes. Telomere length (TL) shortening is associated with age and many other risk factors of stroke. Consistently, a number of studies indicated that stroke occurrence is associated with short leukocyte telomere length (LTL) (2–4). Moreover, accumulated evidence indicates that short LTL is also associated with the post-stroke recovery. Therefore, we aimed to summarize the association between LTL and stroke, and discuss the potential of TL as a biomarker to predict the risk and prognosis of stroke.

## TL Maintenance

### Telomeres

**Figure 1:**
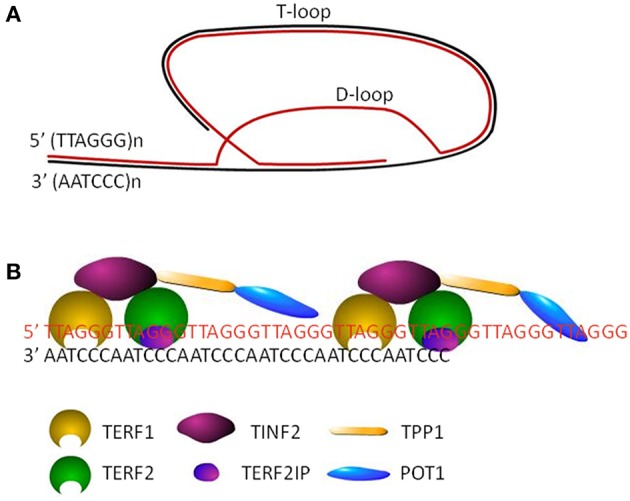
The structure of telomeres. **(A)** Cap structure of telomeres consists of a T-loop and a D-loop. **(B)** The shelterin complex, including TERF1, TERF2, TINF2, TERF2IP, TPP1, and POT1, maintains the stability of loop structures by binding to the telomeric DNA.

Human telomeres are specialized structure of DNA-protein complex, capping the end of linear chromosomes. Human telomeric DNA consists of a variable number of tandem repeats of double-stranded TTAGGG and a 3′ G-rich single-stranded overhang, known as the G-tail (5). The length of double-stranded repeats ranges from 2 to 30 kb, while the length of 3′ overhang is around 150 nt. The invasion of 3′ G-tail into the double-stranded region forms a high order structure and a triple-stranded structure, called T-(telomere) loop and D-(displacement) loop, respectively (6) (Figure 1A). Shelterin complex, the protein components of telomere, includes six subunits, telomeric repeat binding factor 1 (TERF1), telomeric repeat binding factor 2 (TERF2), TERF2 interacting protein (TERF2IP), TERF1-interacting nuclear factor 2 (TINF2), tripeptidyl peptidase I (TPP1), and protection of telomeres 1 (POT1) (Figure 1B). TERF1/TERF2 and POT1 bind directly to double-stranded and single-stranded telomeric DNA, respectively (Figure 1B). TERF2IP, the partner of TERF2, interacts solely with TERF2, while TINF2 connects TERF1, and TERF2. POT1 is tethered to TERF1 and TERF2 via an interaction between TPP1 and TINF2 (7). Telomeric DNA along with the shelterin protein complex maintains the specialized telomere structure and protects the ends of chromosomes from degradation, fusion, and recombination for maintaining genomic integrity (8). Moreover, mammalian CTC1-STN1-TEN1 (CST) complex does localize to telomeres, which resembles the Cdc13-Stn1-Ten1 complex from *Saccharomyces cerevisiae* in that the STN1 and TEN1 subunits are conserved. CST complex binds to single strand DNA in a non-sequence specific manner and plays a key role in telomere replication (9–11). In addition, various DNA repair proteins contribute to telomere maintenance including TL maintenance via interacting with telomere proteins or modifying telomere proteins, including Polβ, FEN1, APE1, XPF-ERCC1 complex, and MRN complex which are involved in base excision repair pathway, nucleotide excision repair pathway and double strand break repair pathway, respectively (12–15).

### Telomerase

Inability of DNA polymerase complex to replicate the 3′-end of the lagging strand in linear chromosomes leads to telomere shortening by each cycle of DNA replication during cell division, so-called end replication problem. As the number of cell division increases, TL is gradually shortened. When TL is reduced to the critical length, cells stop dividing and may enter cellular senescence or apoptosis (16). To counter this issue, human telomerase is responsible for synthesizing telomeric DNA to compensate the erosion of TL during DNA replication. Telomerase is a unique ribonucleoprotein enzymatic complex consisting of a RNA component and a protein component, telomerase RNA component (TERC), and telomerase reverse transcriptase (TERT). TERC is a non-coding RNA which is essential for telomere synthesis by serving as a template for the elongation of telomere 3′ overhang of the telomeric G-rich strand, while TERT catalyzes the process by its reverse transcriptase activity (17). In mammalian, telomerase is active only in germ cells, some types of stem cells and cancer cells, while most human somatic cells lack telomerase activity. Moreover, the activity of telomerase is regulated by the shelterin complex, CST complex, and telomeric repeat containing RNA (TERRA) which is transcribed from telomeric DNA. For example, shelterin complex control telomerase-dependent telomere elongation through a TERF1-mediated negative regulatory pathway, i.e., the more TERF1 is binding to telomeres, the less telomerase accesses to the ends of telomeres leading to less telomere elongation (13, 18, 19). In addition, the phosphoryaltion and PARylation of TERF1 promotes the release of TERF1 from telomeres contributing to telomere lengthening (13, 20, 21). Recently, the structure of telomerase with telomeric DNA has been resolved, which will promote the understanding the function of telomerase (22).

## TL and Stroke

TL maintenance is critical for human health as telomere shortening is associated with aging and various age-related diseases. Leukocyte telomere length (LTL) is served as a marker in a number of association studies of aging and age-related diseases (3, 23–26). The alteration of LTL has been observed in patients with stroke, which is one of the most common age-related diseases leading to death and disability. The positive association between short LTL and the risk of stroke was reported in the case-control studies from China, while the short LTL was also associated with post-stroke death (2, 25, 27, 28). In a 10-year prospective study from the United States, the positive association between short LTL and increased risk of stroke was only existed in the population ranging from 65 to 73 years old, but not in the population beyond 73 years old (29). A study from Europe showed that short LTL was associated with cerebrovascular accidents in the hypertensive patients with left ventricular hypertrophy aged 55–80 years (30). Moreover, short LTL was associated with higher mortality rate in patients with cardiovascular diseases aged 60 years or older (31). However, two nested case-control studies from the United States have not found the positive association between LTL and stroke (32, 33). In addition, no association between short LTL and stroke was found in a 29-year cohort study from Denmark (34). It has to be noted that the participants aged 30–55, 40–84, and 30–60 years, respectively, in the three studies when the blood samples were collected. It highly suggested that the positive association between short LTL and the risk of stroke or post-stroke death might only exist in the aged population although further validation is necessary. Recently, two meta-analysis have been performed to investigate the association between TL and stroke. First, a meta-analysis including 11 studies showed that shortened TL is significantly associated with stroke, even though subgroup analysis showed equivocal results for both prospective (*n* = 7, *p* = 0.051) and retrospective (*n* = 4, *p* = 0.067) studies (35). The high heterogeneity, particularly age difference, and reduced sample size might contribute the equivocal results of the subgroup analysis. Another meta-analysis based on causal inference approaches using TL-related SNPs as instrumental variables did not support the causal effect of shorter TL on ischemic stroke (36). It has to be noted that age, one of the most important covariates, was not considered in this study. As previous studies do indicate that the positive association between short LTL and the risk of stroke or post-stroke death might only exist in the aged population, the effect of age might need to be taken into consideration in future studies. Currently, although mechanisms of the positive association between short LTL and stroke in the elderly remain elusive, multiple signaling pathways may contribute the positive association, e.g., apelin-apelin receptor mediated signaling pathway, histone deacetylases mediated epigenetic regulation (37, 38).

## Risk Factors of Stroke and TL

### Age

Age is one of the major risk factors of stroke (39). The incidence of stroke is increased with age, and the mean age at first-ever stroke was more than 68 years (40). TL is considered as a marker of the biological aging process because it shortens with each cell division. The estimated shortening rate is about 25–27 base pairs per year with individual difference (23, 41, 42). However, the actual LTL shortening rate is about 31–72 base pairs per year (23, 43). It suggests that many factors may contribute to LTL shortening in addition to end replication problem. For example, oxidative stress-induced oxidative DNA damage could lead to telomere uncapping and subsequent telomere shortening, while telomere shortening further enhances oxidative stress forming a vicious cycle (44–47). Age-related increase of oxidative damage is observed in human (48, 49). Moreover, increased evidence suggests that reduction of mitochondria function contributes to the aging process (50). Dysfunction of mitochondria contributes to age-related increase of oxidative stress, which in turn leads to TL shortening (51). Furthermore, multiple molecules (e.g., apelin, HDAC4, RCAN1, α-synuclein), altered in stroke are implicated in aging process, premature disorders (e.g., Down syndrome, Werner syndrome) and age-related disorders (e.g., Alzheimer's disease, Parkinson's disease, Huntington's disease) (37, 38, 52–66). Thus, age-related physiological or pathological alterations may also contribute to TL shortening.

### Genetic Factors

Growing evidence indicates that genetic factors play a major role in stroke and family history of stroke is associated with increased stroke risk (67–69). Genomewide association study (GWAS) showed that the heritability for all ischemic stroke was 37.9%, while the heritability varied markedly in different subtypes of stroke, 32.6% for cardioembolic stroke, 40.3% for large-vessel stroke and 16.1% for small-vessel stroke (70). It was also reported that genetic factors appeared to be more important in large-vessel and small-vessel strokes than in cardioembolic stroke (71). Moreover, various genetic variants associated with short telomeres also contribute to the risk of stroke. For example, genetic variants of *ACYP2* and *TSPYL6* were associated with shorter telomere, which did contribute to the increased risk of stroke (72, 73).

### Obesity

Obesity is associated with elevated risk of stroke. A number of studies showed that obesity is consistently associated with increased inflammation and oxidative stress, which promotes telomere shortening (74–80). Valdes et al. found that LTL of obese women was 240 bp shorter than that of age-matched lean women (41). A couple of cross-sectional studies showed a negative correlation between LTL and obesity (81, 82), whereas others did not show significant associations between LTL and obesity (83, 84). A recent meta-analysis showed there is a tendency toward demonstrating negative correlation between obesity and TL. Although 39 of 63 studies showed either weak or moderate correlation between obesity and TL, heterogeneity among the selected studies made the relationship of obesity and TL still open (85).

### Smoking and Alcohol Intake

Smoking is a well-established risk factor for all forms of stroke (86–88). Even passive smoking significantly increases the risk of stroke (89–91). Meta-analysis including 30 studies showed that shorter LTL was among ever smokers compared to never smokers, while the dosage of smoking was negatively associated with LTL (92). Moreover, Valdes et al. showed that each pack-year smoked caused an additional loss of 5 base pairs of LTL per year (41). Babizhayev et al. reported that telomere attrition can serve as a biomarker of the oxidative stress and inflammation induced by tobacco smoking (93). In addition, heavy alcohol consumption was strongly associated with a reduced LTL in participants aged ≥65 years (94).

### Psychological Stress and Depression

Numerous studies demonstrated that psychological stress caused by negative life event, work stress, illness etc. has significant effect on the risk of stroke (95–98). Accelerated LTL shortening was associated with psychological stress, which might be caused by psychological stress-induced higher oxidative stress and lower telomerase activity (99, 100). In addition, depression is a strong risk factor for stroke, while long-term stress could lead to depression (101, 102). Although the association between LTL and depression was inconsistent, two meta-analyses showed that short LTL was associated with depression (103, 104).

### Age-Related Diseases

#### Hypertension

Hypertension is one of the most important modifiable risk factors for stroke. The strong, direct, linear, and continuous association between increased blood pressure and stroke risk was observed, while intensive blood pressure lowering appears to be beneficial for the reduction of stroke incidence (105–109). Two studies showed that LTL was negatively associated with pulse pressure and hypertension (110, 111). Importantly, meta-analyses from 3,097 participants showed that LTL of hypertensive patients was significantly shorter than that of controls (112).

#### Diabetes

Diabetes is an independent risk factor for stroke with a 2-fold increased risk in stroke for diabetic patients (113). Compared with control individuals, TL is significantly shorter in patients with type 1 diabetes or type 2 diabetes (114–121). For example, LTL was significantly shorter in type 2 diabetes cases compared with controls over a wide age range in a retrospective case-control study comprising 4,016 subjects (121). Moreover, the escalated telomere attrition was associated with insulin resistance in young adults aged from 21.0 to 43.5 years, which might be caused by increased oxidative stress, metabolic and inflammatory factors (122). In addition, the inverse association between LTL and body mass index (BMI)was observed (120).

#### Cardiovascular Diseases

Short LTL are associated with various cardiovascular diseases (CVD) including atherosclerosis, myocardial infarction, coronary artery disease (CAD) and atrial fibrillation (AF) (123–129). For example, short TL was associated with severe CAD adjusting for age and sex (123). A recent systematic review and meta-analysis from 43,725 participants and 8,400 patients suggested that LTL was inversely associated with the risk of coronary heart disease independent of conventional vascular risk factors (26). LTL was shorter in patients with myocardial infarction than that in age-matched control individuals (124). In a prospective study, Chen et al. examined LTL at baseline to predict the incidence and progression of carotid atherosclerosis (130). Compared to participants in the highest LTL tertile, those in the lowest tertile had significantly elevated risk for both incident plaque and plaque progression (130). A recent study indicated that telomere shortening may constitute a novel factor for cardioembolic stroke in patients with AF (129).

Although the underlying mechanism of the relationship between short LTL and CVD remains elusive, booming studies have provided a number of hypotheses (131–133). First, genomic evidence showed that inheritance of shorter telomere might act as a cause of CVD. Genome-wide association studies (GWAS) identified a small set of loci, including SNPs within *TERT* and *TERC*, but all with very small *R*^2^ values (<0.5%) (72, 134). Moreover, it has been reported that cardiovascular risk-related systemic oxidative stress and inflammation might accelerate telomere attrition (135). In addition, the attrition-rate hypothesis implies that variation in adult telomere attrition rates might be more important than TL inheritance (136), indicating that telomere attrition rate could serve as a clinical atherosclerosis CVD biomarker. However, a smaller longitudinal study did not show and so on an association between leukocyte telomere attrition rate and atherosclerosis (137).

## Conclusions

Short LTL is positively associated with stroke in the elderly population. In addition, multiple risk factors of stroke are associated with shortened LTL, e.g., age, smoking, alcohol intake, psychological stress, while the possibility of short LTL as an independent risk indicator of stroke still remains. It indicates that LTL might serve as a potential biomarker to evaluate the risk of stroke in the elderly. Moreover, short LTL was also positively associated with the mortality of stroke and cardiovascular diseases, indicating that LTL might be a potential biomarker to predict the prognosis of stroke.

## Author Contributions

YT, SW, FJ, QK, and CL wrote the manuscript. YW formulated and revised the manuscript.

### Conflict of Interest Statement

The authors declare that the research was conducted in the absence of any commercial or financial relationships that could be construed as a potential conflict of interest.
